# A Tunable Catadioptric Spectrometer with Bragg-Condition-Preserving Rotation for High-Resolution Spectroscopy

**DOI:** 10.3390/s26092761

**Published:** 2026-04-29

**Authors:** Zhongyi Yao, Shuoying Ren, Xinbing Wang, Duluo Zuo

**Affiliations:** Wuhan National Laboratory for Optoelectronics, Huazhong University of Science and Technology, Wuhan 430074, China; d202080953@hust.edu.cn (Z.Y.); xbwang@hust.edu.cn (X.W.)

**Keywords:** tunable catadioptric spectrometer, volume phase holographic grating, Raman spectroscopy, transmission spectrometer

## Abstract

**Highlights:**

- A tunable catadioptric spectrometer was developed by integrating a grating–mirror rotating assembly into a transmission-type VPH (volume phase holographic) grating spectrometer.
- Two key characteristics of the optical configuration were derived through theoretical analysis. The design ensures that the radiation of the Bragg wavelength can always reach the optical axis of the imaging lens during wavelength tuning, while the incident and diffracted beams at the grating simultaneously satisfy the Bragg condition.
- The diffraction efficiency of the tunable catadioptric spectrometer was experimentally characterized and found to be relatively high across the tuning range. The spectral resolution was measured to reach 0.1 nm at 626 nm. In addition, the MTF and spot diagram performance of the spectrometer were evaluated through optical simulations, demonstrating good imaging quality.
- The design methodology and operational principles of the spectrometer are described in detail. The proposed system enables wavelength tuning while maintaining either a constant spectral resolution or a constant optical throughput.
- The tunable catadioptric spectrometer was further validated in fiber-enhanced gas Raman spectroscopy experiments using standard gas samples. Overlapping Raman peaks of CH_4_ and C_2_H_6_ were clearly resolved, confirming the high spectral resolution and high optical throughput of the spectrometer.

**Abstract:**

High-throughput and compact volume phase holographic (VPH) grating transmission spectrometers are widely employed in scientific research, agriculture, and industrial applications. Conventional transmission spectrometers generally adopt a fixed configuration and therefore have limitations in simultaneously achieving high spectral resolution and broad wavelength coverage. To address the limited tunability of transmission spectrometers, this work presents the theoretical analysis and experimental validation of a transmission spectrometer incorporating a novel catadioptric grating assembly, which consists of a transmitting VPH and a planar reflector. A catadioptric system is a combination of reflective (catoptric) and refractive (dioptric) elements. In the proposed configuration, a VPH grating and a plane mirror arranged at a fixed 90° angle form the catadioptric dispersion module. Synchronous rotation of this assembly enables wavelength scanning. The structure ensures that the diffracted ray along the optical axis of the imaging lens maintains the Bragg condition across the scanning range, thereby preserving maximum diffraction efficiency. The optical configuration and structural parameters of the spectrometer were theoretically derived, and a prototype spectrometer with an f-number of 1.8 employing a 2400 g/mm grating was constructed. Measurements demonstrate that, when the rotation angle is tuned from 30.5° to 50.5°, the accessible spectral range covers from 410 nm to 650 nm. Spectral response measurements using a tungsten–halogen light source confirm that the spectrometer maintains an acceptable diffraction efficiency across the entire tuning range. The measured spectral resolution is 0.1 nm at 626 nm with a 2400 g/mm grating and 0.18 nm with a 1500 g/mm grating. The spectrometer was further applied to fiber-enhanced gas Raman spectroscopy, where it successfully resolved the closely spaced Raman peaks of CH_4_ and C_2_H_6_ that are difficult to distinguish using conventional compact spectrometers. These results demonstrate that the proposed tunable catadioptric spectrometer simultaneously provides excellent wavelength tunability and high spectral resolution.

## 1. Introduction

Transmission spectrometers are widely used in scientific research, industry [1], and agriculture [2]. A typical transmission spectrometer consists of a collimating lens, a grating, an imaging lens, and an array detector. This configuration provides the advantage of a large numerical aperture. Compared to Czerny–Turner spectrometers, which utilize off-axis reflective optics, transmission spectrometers provide higher throughput and achieve superior imaging quality using refractive optics. These instruments are vital in various fields, such as hyperspectral applications [3], where they are used to observe solar-induced chlorophyll fluorescence of vegetation in the near-infrared band. In laser-induced breakdown spectroscopy applications [4,5], where a pulsed high-power laser irradiates a sample, the spectrum is analyzed to determine the atomic composition of the material. In Raman spectroscopy applications [6], these devices are employed to measure the geological composition of extraterrestrial planets. In some applications, it is necessary to increase the slit width to enhance throughput; however, this inevitably leads to a loss of resolution. To mitigate this, techniques such as virtual slits or coded apertures are often employed [7]. In spectrometer design, a trade-off exists between spectral range and resolution. Consequently, spectrometers used in astronomical observatories often achieve wavelength tuning by simultaneously rotating both the grating and the array detector. However, this approach results in a mechanically complex structure [8].

In conventional LGL (lens–grating–lens) spectrometers, when higher spectral resolution is required, the spectral range is consequently reduced. Moreover, the slit width must be reduced, or the focal length of the lens must be increased, which inevitably decreases the optical throughput of the spectrometer.

This study presents an LGL spectrometer incorporating a rotatable catadioptric grating module for Raman spectroscopy, hereafter referred to as a tunable catadioptric spectrometer, which is applied in our experiments for field Raman gas analysis and Raman micro-spectroscopy. A catadioptric system is a combination of reflective [catoptric] and refractive [dioptric] elements. The tunable catadioptric spectrometer has two main advantages. First, it functions as a tunable spectrometer. For example, the Bragg wavelength of the spectrometer can be tuned over a wavelength range from 410 nm to 650 nm. Second, this design improves spectral resolution and achieves the required spectral range. The spectrometer employs a grating with a higher groove density and acquires the spectrum in two or more frames while maintaining constant throughput. The rotational structure allows the spectrometer to vary its spectral range and enables the use of a single-element detector for wavelength scanning, as opposed to an array detector. This study describes the theoretical design and construction of this spectrometer. Additionally, a prototype with an f-number of 1.8 was built, which is suitable for high-resolution measurements within the visible spectral range.

## 2. Principle of the Tunable Catadioptric Spectrometer

**Figure 1:**
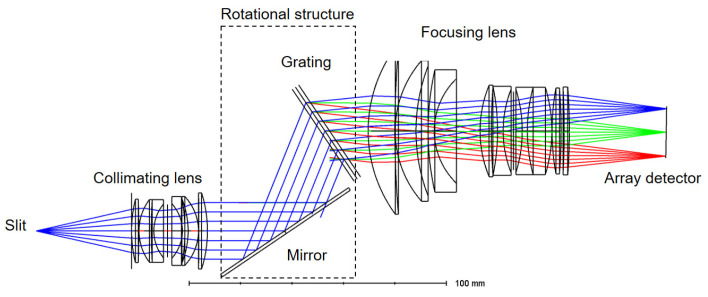
Diagram of the tunable catadioptric spectrometer.

As illustrated in the optical layout in Figure 1, the spectrometer’s structure consists of a slit, a collimating lens, a reflective plane mirror, a VPH grating, an imaging lens, and an array detector. For simplicity, a grating with fringes perpendicular to its planar surface is selected. The intersection of the grating plane and the plane mirror lies on the central line between the parallel optical axes of the imaging lens and the collimating lens.

The VPH grating and the plane mirror, which is perpendicular to the grating plane, constitute the dispersion module of the catadioptric spectrometer, a key module that distinguishes the catadioptric spectrometer from other VPH grating LGL spectrometers.

**Figure 2:**
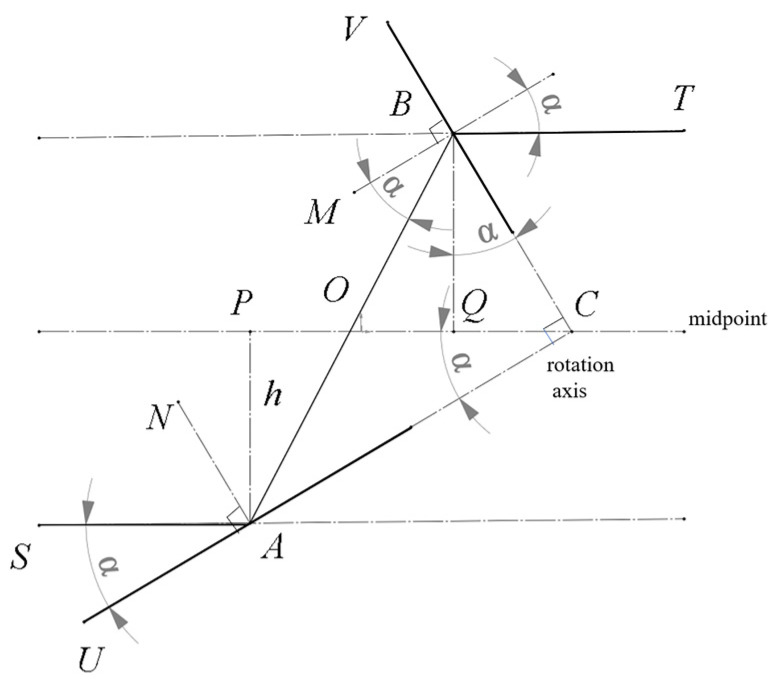
Schematic diagram demonstrating the structural principle of the tunable catadioptric spectrometer.

The tunable catadioptric spectrometer exhibits two key features: invariant optical axis position and angular compliance. The position characteristic of the spectrometer structure means that the radiation of the Bragg wavelength in the incident beam to the VPH grating can always reach the optical axis of the imaging lens, regardless of how the structure rotates, and the angular compliance means the diffracted beam along the optical axis of the imaging lens is just the diffraction direction of the Bragg wavelength. The grating and the mirror remain perpendicular and rotate around an axis. This axis is also a midpoint and lies on the line parallel to the optical axes of the two lenses. The derivation is as follows: The geometry relation in the grating module is shown in Figure 2, where AN and BM are the normal to the mirror and grating plane, respectively; CQ is a symmetrical axis parallel to SA which is the axis of the collimating lens or the central line of the incident beam; AP and BQ are perpendicular lines to CQ; and α is the angle between the incident ray SA and the mirror. As the grating plane is set perpendicular to the mirror, we have ∠CBA=∠BAN=π/2−α=∠BCQ, and ∠ABM=α; thus, OA=OB=OC, and BQ=AP=h is a constant value regardless of the variation in angle α.

The angular characteristic is demonstrated below. When BT∥CQ, the angle between BT and the normal BM of the grating will also be α, which means BT will be the diffraction ray of the Bragg wavelength. By positioning the rotation axis at point C, rotating the grating module around point C will change the Bragg angle α and the spectral range recorded by the array detector behind the imaging lens.

There are several advantages to the catadioptric spectrometer when compared with a Czerny–Turner spectrometer. Lenses are used for collimating and imaging in the catadioptric spectrometer, and as all the rays are near paraxial, a larger NA (lower F#) is possible. The NA of a Czerny–Turner spectrometer is relatively small. In terms of overall size, the catadioptric spectrometer can also be made much more compact. Unlike the reflective gratings used in a Czerny–Turner spectrometer, VPH gratings exhibit high diffraction efficiency, low stray light, high achievable groove density, and large aperture, which makes them suitable for astronomical spectrometers.

**Figure 3:**
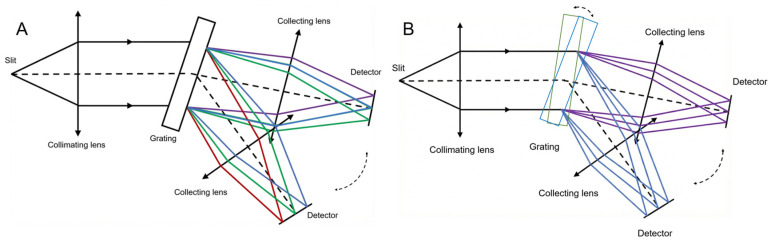
Two rotational designs of tunable spectrometers. (**A**) shows the first design, in which the camera system is rotatable. (**B**) shows the second design, in which both the grating and the camera system are rotatable.

To address the trade-off between spectral range and spectral resolution, several studies have proposed rotating multi-structure spectrometer designs, as illustrated in Figure 3. In the configuration of Figure 3A [8], the collection lens and the detector are rotated simultaneously. However, a major drawback of this approach is that the incident angle and diffraction angle at the grating along the axis of the imaging lens do not satisfy the Bragg condition, resulting in a reduction in diffraction efficiency.

A design has been adopted in astronomical telescope spectrometers to overcome the limited spectral coverage associated with high-resolution observations, which requires the simultaneous rotation of both the grating and the camera system, each rotating at a different angle. The optical path satisfies the Bragg condition, as shown in Figure 3B [9]. While effective, such a design leads to increased structural and mechanical complexity. In contrast, the design proposed in our work differs fundamentally from both approaches. Only a combined grating–mirror assembly is rotated, which significantly reduces the number of moving components. Moreover, the diffracted ray along the axis of the imaging lens satisfies the Bragg condition throughout the tuning process, thereby maintaining high diffraction efficiency. This design preserves the high-resolution advantage of a fixed camera system composed of the lens group and the array detector, while simultaneously offering a simpler mechanical structure and improved diffraction efficiency.

## 3. Design of the Tunable Catadioptric Spectrometer

### 3.1. Design of a Spectrometer for Higher Raman Spectral Resolution

Raman spectroscopy represents an important application direction for LGL spectrometers. The commonly used Raman shift range is 200–4000 cm^−1^. The design parameters of a tunable catadioptric spectrometer with a grating of 2400 g/mm and a conventional Raman spectrometer with a grating of 1800 g/mm are listed in Table 1. Higher resolution is obtained by the high-density grating for the catadioptric spectrometer. To cover the full Raman shift range, the catadioptric grating module is set at 2 different angular positions. The mirror for the catadioptric grating module should accommodate the large incident angle range and the wavelength range of 538–676 nm. In addition, the two lenses should also be suitable for this wavelength range, which can be readily satisfied by camera lenses. The grating incidence angle for the conventional Raman spectrometer can be calculated by the Bragg condition of the center wavelength, which is α = 33°. The collimating lens has an NA of 0.25 and a focal length of 50 mm. The focal length of the imaging lens can be calculated based on the relationship between the detector’s image size and the diffraction angles of each wavelength.

(1) $$\theta =\sin^{-1}(\frac{\lambda }{d}-\sin\theta_{B})$$

(2) $$f_{2}[\tan(\theta_{\max}-\theta_{B})+\tan(\theta_{B}-\theta_{\min})]=L$$

where L is the image width along the dispersion direction (L = 25.6 mm). L is slightly smaller than the illuminated length of the CCD to allow for a margin. d is the grating pitch, $\theta_{B}$ is the Bragg angle, θ is the diffraction angle for wavelength λ, and $\theta_{max}$ and $\theta_{min}$ are margins for the diffraction angles. The diffraction angle at 538 nm is 25.1°, and the diffraction angle at 676 nm is 42.2°, resulting in a required field of view of 42.2° − 25.1° = 17.1° for the imaging lens. The imaging lens has a diameter of 43.9 mm and a focal length of *f*_2_ = 85 mm, leaving some margin for the detector.

When the slit width is 10 µm, the spectral resolution is determined by the pixel width. The resulting resolution is 6 cm^−1^, which is a calculated value.

The design of the tunable catadioptric spectrometer is described as follows: If the required Raman-shift range can be acquired in several frames, a grating with a higher groove density can be applied. If an 1800 g/mm grating is selected for the catadioptric spectrometer, the full Raman shift range can be covered by 1 frame. If we replace the grating with a 2400 g/mm one while keeping all other hardware unchanged, the same spectral range can be recorded using 2 frames. In Frame A, the spectral range is 200–2800 cm^−1^, corresponding to 538–625 nm, and the diffraction angle for the center wavelength of 581 nm is 44.2°, at which the Raman resolution is improved to 4.2 cm^−1^.

In Frame B, the spectral range is 2100–4000 cm^−1^, covering a wavelength range from 599 nm to 676 nm. The diffraction angle at the center wavelength of 638 nm is 50°, at which the Raman resolution is improved to 3.1 cm^−1^. At 605 nm, the resolution is 3.4 cm^−1^.

**Figure 4:**
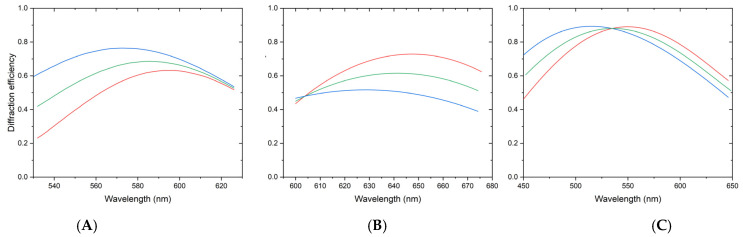
Simulated diffraction efficiency curves of the 2400 lines/mm grating: (**A**) Frame A, (**B**) Frame B. The red, blue, and green curves correspond to s-polarization, p-polarization, and the average value, respectively. (**C**) shows the efficiency curve of the 1800 g/mm grating [10].

When the required Raman-shift range is acquired in one shot with an 1800 g/mm grating, the spectral resolution is 6 cm^−1^. The resolution improves to 3.4 cm^−1^ when a higher-groove-density grating is used, and the spectral range is divided into two frames. As shown in Figure 4, the diffraction efficiency of the grating does not decrease significantly due to tuning. When using an 1800 g/mm grating, the diffraction efficiency can reach nearly 90% at its peak.

Compared with a tunable reflective spectrometer, the LGL spectrometer has a much larger NA, and its sensitivity can reach up to several times that of the reflective spectrometer. Although the diffraction efficiency of the VPH grating decreases, the tunable LGL spectrometer still maintains high sensitivity due to the inherently high transmittance of the VPH grating.

In addition, increasing the focal length of the spectrometer can also improve its spectral resolution. When the focal length is increased, the resolution improves; however, assuming the physical aperture of the lens remains unchanged, the collection NA becomes smaller, resulting in reduced spectrometer throughput. When detecting weak light sources, a high-throughput spectrometer is required, and therefore, the method of increasing the focal length is not recommended.

### 3.2. Spectral Resolution of the Tunable Raman Spectroscope with Different Exciting Wavelengths

**Figure 5:**
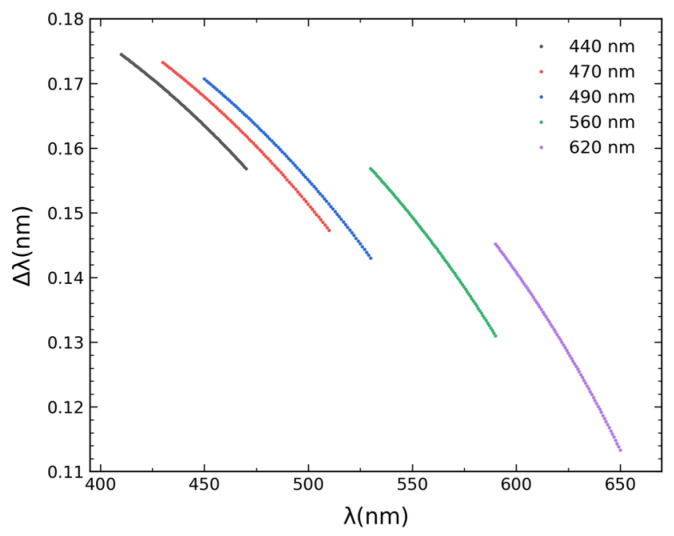
Spectral resolution over the full span when the center wavelength of the spectrometer is set at 440 nm, 470 nm, 490 nm, 560 nm, and 620 nm.

The tunable catadioptric spectrometer can be applied to Raman spectroscopy with multiple excitation wavelengths, such as 410 nm, 450 nm, and 532 nm. The corresponding detection wavelength ranges are 410–465 nm, 456–526 nm, and 540–641 nm, respectively. By rotating the grating of the spectrometer, the center wavelength is tuned to 440 nm, 470 nm, 490 nm, 560 nm, and 620 nm. The spectral resolution over the full span was calculated based on a two-pixel criterion, and the corresponding curves are shown in Figure 5. The two-pixel resolution should be regarded as a guaranteed minimum value. In the experiments, the spectral profile can often be fitted with a width of about 1.5 pixels using a CCD with a pixel size of 20 μm. When the spot diameter is slightly smaller than two-pixel widths, adopting a two-pixel criterion is reasonable and consistent with the Nyquist sampling criterion. By contrast, when the spot diameter extends over multiple pixels, it is more appropriate to define the resolution in terms of the FWHM.

### 3.3. Simulation of the Spectrometer

**Figure 6:**
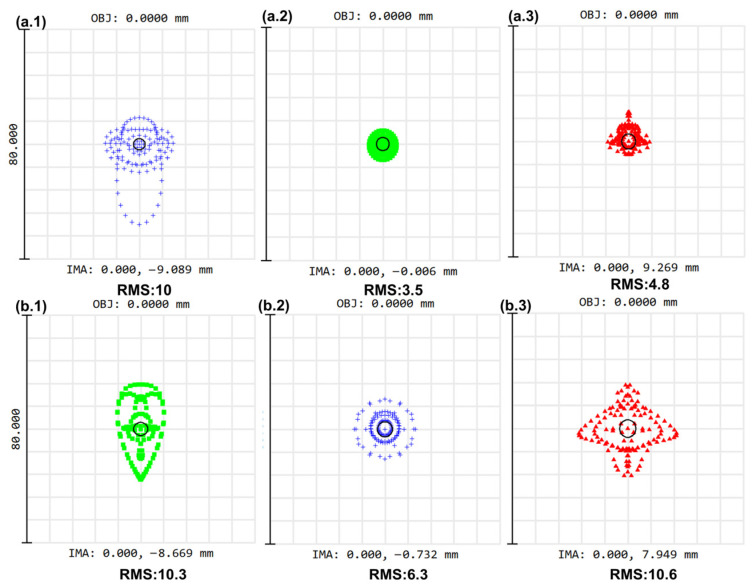
Spot diagrams at 430 nm, 470 nm, and 500 nm [(**a.1**–**a.3**)] and spot diagrams from a separate test at 530 nm, 560 nm, and 590 nm [(**b.1**–**b.3**)].

**Figure 7:**
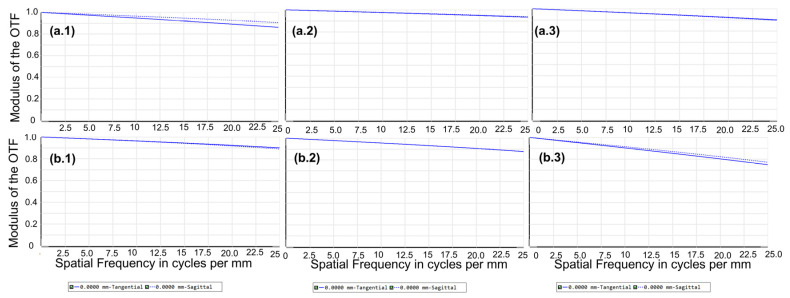
MTF curves at 430 nm, 470 nm, and 500 nm [(**a.1**–**a.3**)] and those from a separate test at 530 nm, 560 nm, and 590 nm [(**b.1**–**b.3**)].

A tunable catadioptric spectrometer prototype was designed for applications within the visible spectral range, in which commercial off-the-shelf lenses were utilized. As shown in Figure 6, the imaging performance of the spectrometer was subsequently characterized through simulation, with an assumption of NA = 0.22 for the input radiation. In the 430–500 nm range, the RMS spot size is less than 10 µm, which is smaller than the detector’s pixel size of 20 µm. Similarly, in the 530–590 nm range, the RMS spot size remains within 11 µm. As shown in Figure 7, the Nyquist frequency of the detector is $1/2l_{pix}=25$ lp/mm. In both spectral ranges, the modulation transfer function (MTF) values for all wavelengths exceed 0.6. These results indicate effective aberration correction and high imaging quality. However, in the 590–670 nm range, the simulated imaging performance is suboptimal, suggesting that the lens design requires further optimization [11,12]. The lenses used in this work are commercial photographic lenses with built-in aberration correction. In practice, their imaging performance is reasonably good, and the Raman spectrometer can achieve the designed spectral resolution over the 540–670 nm range. If further optimization is required, the distance between the lens and the CCD can be adjusted.

Tolerance errors can cause the beam to become misaligned with the optical axis of the focusing lens, resulting in reduced optical throughput and degraded imaging quality. However, the focusing lens is an 85 mm lens with F# = 1.4 and a very large aperture. In addition, both the collimating lens and the focusing lens are focus-adjustable, which helps compensate for tolerance-induced misalignment.

## 4. Experimental Testing of the Tunable Catadioptric Spectrometer

### 4.1. Spectral Resolution

An experimental prototype of the spectrometer was constructed. The collimating lens has a focal length of 50 mm and an f-number of 1.8, while the imaging lens has a focal length of 85 mm and an f-number of 1.4, and the slit width is 10 µm. The spectral resolution of the spectrometer was experimentally verified. The CCD detector has a format of 2048 × 64 pixels, with a pixel width of 14 µm. In the experiment, a 1500 g/mm grating was first installed in the spectrometer, and the spectral resolution was experimentally evaluated. The grating was then replaced with a 2400 g/mm grating, and the resolution was measured again. The center wavelength was set to 626 nm, and the measurements were performed using a Ne spectral calibration lamp (6032, Oriel Instruments, Stratford, CT, USA).

**Figure 8:**
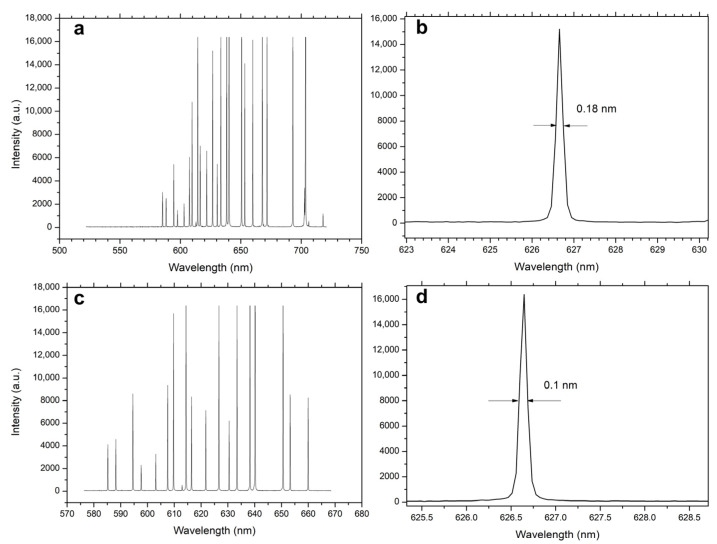
Neon lamp spectra and corresponding FWHM at the central wavelength for 1500 lines/mm and 2400 lines/mm gratings. (**a**) Full spectral range of the 1500 lines/mm grating; (**b**) Magnified view at 626 nm corresponding to (**a**); (**c**) Full spectral range of the 2400 lines/mm grating; (**d**) Magnified view at 626 nm corresponding to (**c**).

Prior to the experiment, calculations were performed, showing that the spectral resolution is determined by the pixel width. A resolution corresponding to two-pixel widths is taken as the spectral resolution. With the 1500 g/mm grating, the calculated resolution is 0.19 nm (4.9 cm^−1^) at 626 nm, while the experimental result is 0.18 nm (Figure 8b). With the 2400 g/mm grating, the calculated resolution is 0.096 nm (2.3 cm^−1^), and the measured result is 0.1 nm (Figure 8d). The measured linewidth in Figure 8 is smaller than the calculated value, which can be attributed to the non-uniform distribution of photons across the two pixels. Diffraction and aberrations must also be considered. Calculations show that the impact of diffraction is negligible. Experimental observations confirm that the presence of aberrations does not prevent the spectrometer from achieving the designed resolution. These results show that, without changing other conditions, replacing the grating improves the spectral resolution, which validates the resolution-enhancement capability of the tunable catadioptric spectrometer. Replacing gratings is common in long-focal-length spectrometers; however, in spectrometers with large numerical aperture, aberrations and other factors must also be considered. Therefore, this experiment provides meaningful validation.

### 4.2. Spectral Response During Spectrometer Tuning

In the testing of spectral response (radiometric response), a grating with a groove density of 2400 g/mm and a CCD detector with a format of 1340 × 100 pixels and a pixel width of 20 μm were applied. The applied grating is an existing component optimized for a Bragg wavelength of 466 nm and is not custom-designed for this experiment.

The spectrometer was tuned to 50.5°, 48.5°, 46.5°, 44.5°, 42.5°, 40.5°, 38.5°, 36.5°, 34.5°, 32.5°, and 30.5°, and spectra were measured at each setting, corresponding to a total wavelength range of 380–670 nm. First, the Ne lamp was used to make the spectral calibration. Second, a tungsten–halogen light source was used to investigate the radiometric response of the spectrometer. For the response measurement, the tungsten–halogen light source (DH2000, Ocean Optics, Dunedin, FL, USA) was coupled into an optical fiber, and the fiber was connected to the tunable catadioptric spectrometer. The transmitted intensity spectrum was measured and then divided by the radiometric calibration data, yielding the response curve.

**Figure 9:**
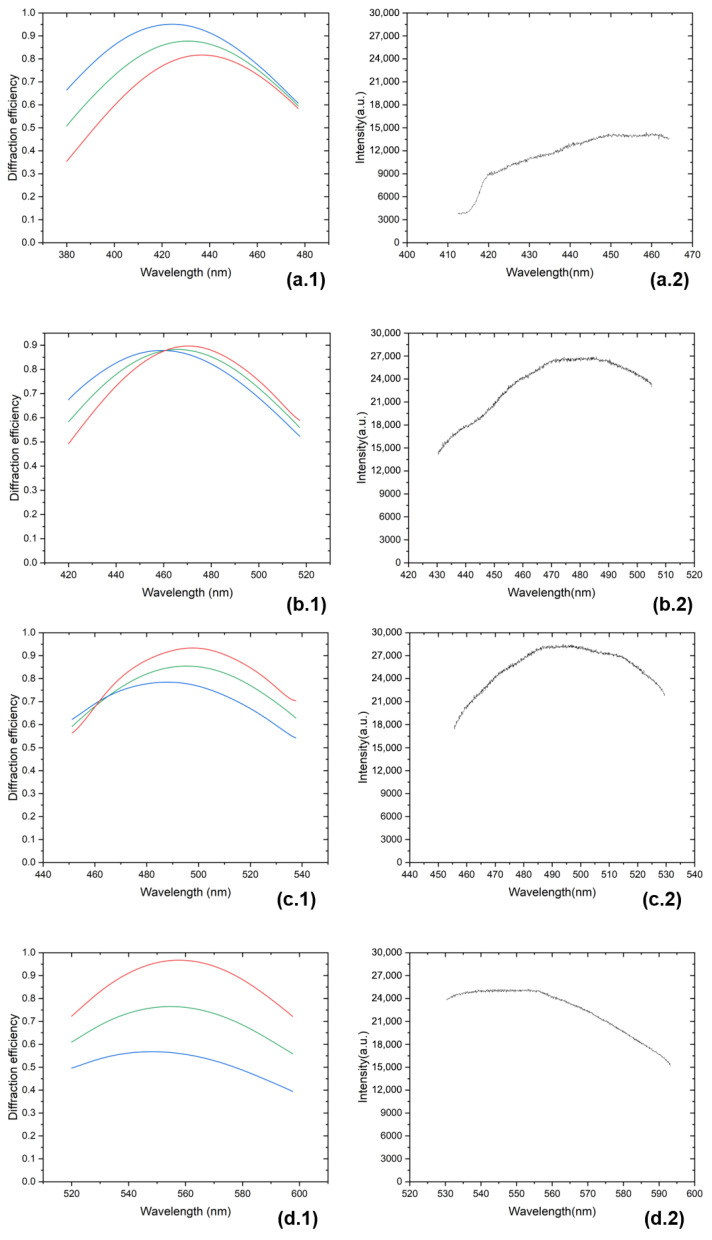

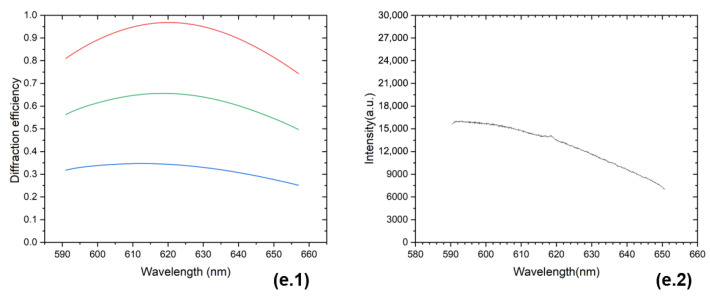
Simulated grating diffraction-efficiency curves and measured spectrometer-efficiency curves at angles of 30.5°, 34.5°, 36.5°, 42.5°, and 48.5°, corresponding to panels (**a.1**–**e.2**), respectively. The red, blue, and green curves correspond to s-polarization, p-polarization, and the average value, respectively.

When the excitation wavelengths are 410 nm, 450 nm, and 532 nm, the corresponding detection wavelength ranges are 410–465 nm, 456–526 nm, and 540–641 nm, respectively. The diffraction responses at rotation angles of 30.5°, 34.5°, 36.5°, 42.5°, and 48.5° are shown in Figure 9.

Some measured response curves deviate from the predicted theoretical curves. In Figure 9(a.2,d.2–e.2), the transmission efficiency curves exhibit a monotonic increase or decrease, without any peak. The experimental results do not agree with the results predicted under the Bragg condition. The discrepancy may arise from the antireflection coating of the grating, which does not meet the requirements for broadband and wide-angle performance. An unmatched antireflection coating can lead to increased reflection for wavelengths outside its design range. In this work, a 600 μm multimode optical fiber (Ocean Optics, Dunedin, FL, USA) and a 10 μm slit were used. This type of fiber does not affect the polarization state of the light and therefore does not influence the polarization-dependent diffraction efficiency of the grating. However, regarding the angular performance of VPH, Samuel Barde and co-workers have studied rotating spectrometers, showing that the grating can operate over a considerably large range of incident angles [13]. When the measured spectral range was in the band of the antireflection coating, as shown in Figure 9(b.2–c.2), the experimental results were in good agreement with the simulation.

As shown in Figure 9(a.2), the transmittance drops sharply at wavelengths below 420 nm. This is attributed to the use of a bandpass filter with a passband of 400–700 nm. The purpose of using the filter is to suppress interference from light outside the target wavelength range.

The grating performance was simulated using Kogelnik’s coupled-wave theory, incorporating a groove density of 2400 g/mm, a refractive index modulation of 0.101, and a grating thickness of 2.45 µm. As shown in Figure 9(b.1,b.2), the designed center wavelength is 466 nm. Figure 9(d.1–e.2) illustrate a pronounced discrepancy between the diffraction efficiencies of s-polarized and p-polarized light. In conventional VPH gratings, p-polarized light is significantly attenuated when the angles of incidence (AOI) and diffraction (AOD) exceed 36°. To address this, a Dickson grating can be employed, which enables high diffraction efficiency for both s- and p-polarizations simultaneously by optimizing the grating thickness and refractive index modulation. Alternatively, an HD grating, known for its wide spectral bandwidth, may be utilized [14].

Certain VPH gratings retain high diffraction efficiency during wavelength tuning by grating rotation, as illustrated below. The grating can be continuously tuned within the 400–700 nm range, maintaining a peak transmission efficiency exceeding 80% with minimal difference between the diffraction efficiencies of p- and s-polarized light. While this tunability is applicable to a 1200 g/mm grating, further research is required to design tunable gratings with higher groove densities [15,16].

The results indicate that the spectrometer exhibits good spectral response in the 410–650 nm range, demonstrating that the rotating spectrometer can operate as a Raman spectrometer operating with excitation wavelengths of 410 nm, 450 nm, and 532 nm.

### 4.3. Verification of the Spectrometer’s Tuning Capability

**Figure 10:**
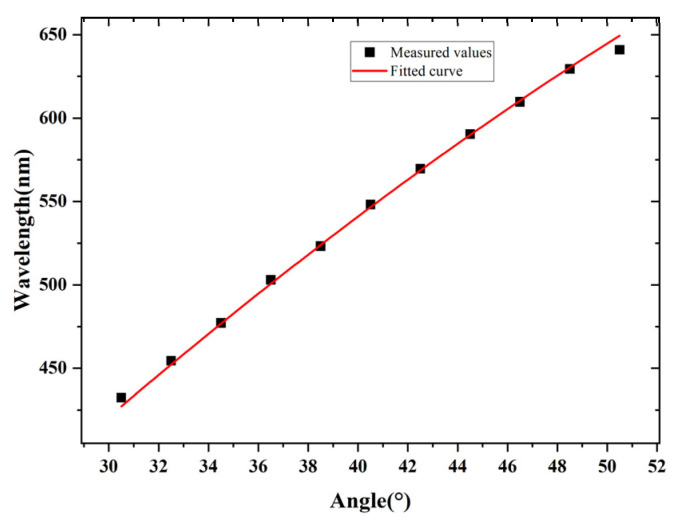
Comparison between the measured wavelengths and the fitted values.

In the experiment, the tunable catadioptric spectrometer was tuned to different center wavelengths. According to our design, the relationship between wavelength and angle should satisfy the Bragg condition. The experimental results are shown in Figure 10. As shown in Figure 10, the wavelength $\lambda_{c}$ measured along the spectrometer optical axis is consistent with the calculated value:

(3) $$\lambda_{c}=2d\sin\theta_{B}$$

where $\theta_{B}$ is the Bragg angle (also the incident angle to the VPH grating), and *d* is the grating pitch. The fitting shows good agreement with the data $R^{2}=0.9977$, indicating that the model describes the relationship between wavelength and angle very well. The residuals are randomly distributed around zero with no obvious systematic trend, suggesting that the error is small and the overall fitting is reliable.

**Figure 11:**
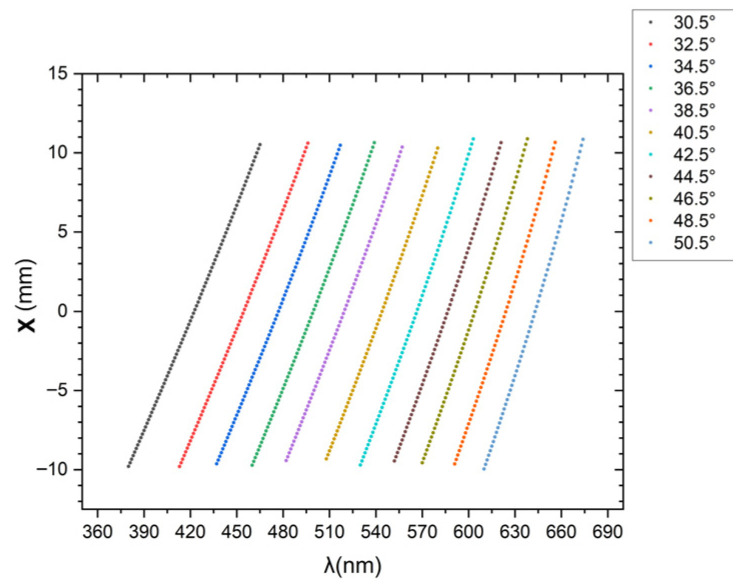
Relationship between spectral range and tuning angle for each step.

As the angle changes, the center of the detected spectrum shifts, and the spectral span also changes accordingly. The calculated variation in spectral span according to Equation (2) and the grating equation, with the assumption of interference order m=1 and detector image width L=20 mm, is shown in Figure 11. The measured spectral span for the tuning angle (also the incident angle or the Bragg angle) of 30.5° is 380–465 nm, with a bandwidth of 85 nm, and that for the tuning angle of 50.5° is 610–674 nm, with a bandwidth of 64 nm, which is consistent with the calculation results of Figure 11. Figure 10 and Figure 11 illustrate the tuning capability of the spectrometer.

Furthermore, as the grating assembly rotates, the position of the beam footprint on both the reflecting mirror and the grating shifts. The light spot shifts by 22 mm on the mirror and 13 mm on the grating, and it is necessary to increase the surface areas of the reflecting mirror and the grating. The minimum width of the reflecting mirror is the sum of the spot displacement and the projected width of the incident aperture. The same applies to the grating.

At the same time, lens vignetting must be considered when designing the spectral range of the spectrometer. Our tungsten–halogen light source measurements show a pronounced decrease near the edges of the efficiency curve, which is caused by lens vignetting. Therefore, optical simulation is required to perform ray tracing and determine whether any rays are blocked by the lenses, and the unvignetted region can be defined as the usable spectral range.

An error analysis of the rotational structure was performed. According to the calculation, when the angular error is 30″, the corresponding wavelength deviation is 0.1 nm, which imposes a stringent requirement on the rotational accuracy. This requirement can be satisfied by using a servo motor with sufficient rotational accuracy.

## 5. Application

**Figure 12:**
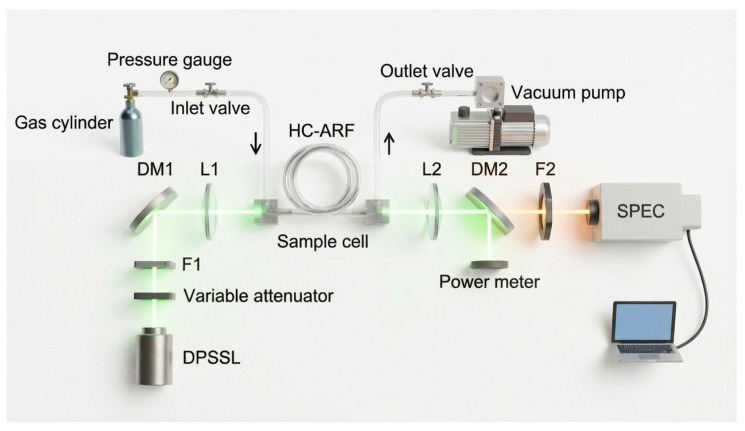
Schematic diagram of the fiber-enhanced gas Raman experimental setup.

Raman spectroscopy experiments were carried out using a tunable catadioptric spectrometer. The spectrometer configuration was identical to that described in Section 3.3. As illustrated in Figure 12, the experimental setup consists of a fiber-enhanced gas Raman detection system. A 100 mW continuous-wave 532 nm laser was employed to excite Raman scattering from a standard gas mixture, with an integration time of 200 s. The standard gas contained 10 ppm of CO_2_, CH_4_, C_2_H_2_, C_2_H_4_, C_2_H_6_, and H_2_ and was maintained at a pressure of 3 bar. Measurements were performed sequentially using two different spectrometer configurations. The first configuration employed a 2400 g/mm diffraction grating, providing a spectral resolution of 3.4 cm^−1^, while the second configuration used an 1800 g/mm grating with a resolution of 6 cm^−1^.

**Figure 13:**
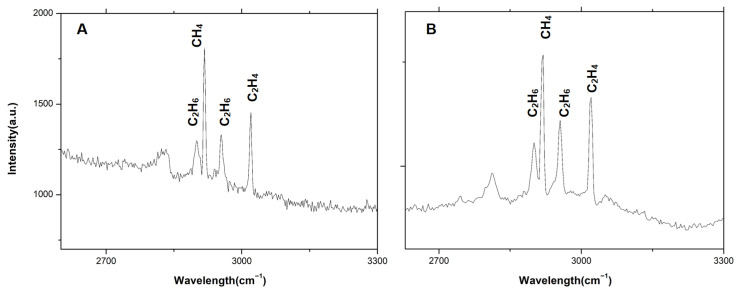
Detection results of the standard gas. (**A**) shows the experimental results obtained using the 2400 g/mm grating, while (**B**) shows the experimental results obtained using the 1800 g/mm grating.

Results are presented in Figure 13A,B. In Figure 13A, obtained with the higher-resolution spectrometer, the Raman characteristic peaks of CH_4_ at 2917 cm^−1^ and C_2_H_6_ at 2900 cm^−1^ in the vicinity of 3000 cm^−1^ are clearly resolved. In contrast, as shown in Figure 13B, these two peaks exhibit noticeable spectral overlap when measured with the lower-resolution configuration. Although peak-fitting algorithms can be used to alleviate peak overlap, acquiring data with higher spectral resolution enables faster and more accurate peak discrimination. These results demonstrate that employing a high-resolution spectrometer effectively reduces spectral overlap among alkane Raman peaks around 3000 cm^−1^ in gas samples. In addition, by adjusting the detection range of the spectrometer, Raman spectra covering the range from 200 to 2800 cm^−1^ can also be obtained.

## 6. Summary

Tunable catadioptric spectrometers are highly desirable because they combine high throughput with a broad spectral range. This study proposes a design methodology for a tunable catadioptric spectrometer and demonstrates its position characteristic and angular invariance. Experiments demonstrate that the spectrometer achieves enhanced spectral resolution while retaining tunability. High spectral efficiency is preserved over a wide wavelength range. During wavelength tuning, the measured center wavelength agrees well with the theoretical predictions. The spectral resolution can reach 0.1 nm at 626 nm. In the simulated imaging performance, the MTF remains higher than 0.6 over the 430–590 nm range. In the Raman experiments, the tunable catadioptric spectrometer can resolve the overlapping Raman peaks of CH_4_ and C_2_H_6_ that are difficult to distinguish using conventional compact spectrometers. This spectrometer therefore offers the dual advantages of tunability and enhanced spectral resolution. Future work will focus on further system validation, as well as continued studies on tunable gratings and imaging quality.

## Figures and Tables

**Table 1:** Parameters for catadioptric and conventional spectrometers.

	Tunable Catadioptric Spectrometer	Conventional Raman Spectrometer
Excitation wavelength	532 nm	532 nm
Wavelength range	538–625 nm (200–2800 cm^−1^) 599–676 nm (2100–4000 cm^−1^)	538–676 nm (200–4000 cm^−1^)
NA	0.27	0.27
Number of exposures	2 frames	1 frame
Image format	1340 × 100 pixels	1340 × 100 pixels
Pixel pitch	20 µm	20 µm
Slit width	10 µm	10 µm
Grating groove density	2400 g/mm	1800 g/mm
Spectral resolution	3.4 cm^−1^	6 cm^−1^
Grating angle	44.2°, 50°	33°

## Data Availability

The data supporting the findings of this study are available from the corresponding author upon reasonable request.
